# Pre-procedural computed tomography predicts procedural complexity and complications in bidirectional rotational mechanical transvenous lead extraction

**DOI:** 10.1093/europace/euaf308

**Published:** 2025-12-06

**Authors:** Federico Migliore, Raimondo Pittorru, Vincenzo Tarzia, Jacopo Rosso, Manuel De Lazzari, Andrea Ziggiotto, Gaia Zancanaro, Giulia Winnicki, Marco Gemelli, Matteo Micciolo, Antonio Guerrieri, Davide Margheri, Raffaella Motta, Valeria Pergola, Gino Gerosa, Domenico Corrado

**Affiliations:** Department of Cardiac, Thoracic, Vascular Sciences and Public Health, University of Padova, Via N. Giustiniani 2, Padua 35121, Italy; Department of Cardiac, Thoracic, Vascular Sciences and Public Health, University of Padova, Via N. Giustiniani 2, Padua 35121, Italy; Department of Cardiac, Thoracic, Vascular Sciences and Public Health, University of Padova, Via N. Giustiniani 2, Padua 35121, Italy; Department of Cardiac, Thoracic, Vascular Sciences and Public Health, University of Padova, Via N. Giustiniani 2, Padua 35121, Italy; Department of Cardiac, Thoracic, Vascular Sciences and Public Health, University of Padova, Via N. Giustiniani 2, Padua 35121, Italy; Department of Cardiac, Thoracic, Vascular Sciences and Public Health, University of Padova, Via N. Giustiniani 2, Padua 35121, Italy; Department of Cardiac, Thoracic, Vascular Sciences and Public Health, University of Padova, Via N. Giustiniani 2, Padua 35121, Italy; Department of Cardiac, Thoracic, Vascular Sciences and Public Health, University of Padova, Via N. Giustiniani 2, Padua 35121, Italy; Department of Cardiac, Thoracic, Vascular Sciences and Public Health, University of Padova, Via N. Giustiniani 2, Padua 35121, Italy; Cardiothoracic Surgery, Bristol Heart Institute, University of Bristol, Bristol, UK; Department of Cardiac, Thoracic, Vascular Sciences and Public Health, University of Padova, Via N. Giustiniani 2, Padua 35121, Italy; Radiology Unit, University of Padua-Azienda Ospedaliera, Padua, Italy; Radiology Unit, University of Padua-Azienda Ospedaliera, Padua, Italy; Department of Cardiac, Thoracic, Vascular Sciences and Public Health, University of Padova, Via N. Giustiniani 2, Padua 35121, Italy; Department of Cardiac, Thoracic, Vascular Sciences and Public Health, University of Padova, Via N. Giustiniani 2, Padua 35121, Italy; Department of Cardiac, Thoracic, Vascular Sciences and Public Health, University of Padova, Via N. Giustiniani 2, Padua 35121, Italy; Department of Cardiac, Thoracic, Vascular Sciences and Public Health, University of Padova, Via N. Giustiniani 2, Padua 35121, Italy

**Keywords:** Computed tomography, Complications, Risk stratification, Rotational mechanical sheaths

## Abstract

**Aims:**

Despite technical advances, transvenous lead extraction (TLE) remains a challenging procedure. Cardiac computed tomography (CT) has emerged as a valuable tool for pre-procedural assessment, but its role in predicting outcomes in rotational mechanical TLE as a first-line strategy is not well defined. The aim was to determine whether pre-procedural CT can predict complications and procedural complexity in patients undergoing rotational mechanical TLE.

**Methods and results:**

This retrospective study included 115 patients. All had pre-procedural contrast-enhanced CT with a dedicated lead extraction protocol. Two procedural outcomes were evaluated: (i) complicated procedure, defined as major complication, incomplete lead removal, or snare use, and (ii) complex procedure, defined as requiring either a snare or a tissue stabilization sheath. Logistic regression and receiver operating characteristic analyses were used to identify predictors. A total of 215 leads were extracted (mean dwelling time 95 ± 73 months). Complicated procedures occurred in 20.9% and were independently associated with longest fibrosis length on CT (odds ratio 1.1; *P* < 0.001); a fibrosis length of >40 mm predicted complicated procedures [area under the curve (AUC) 0.92; 95% confidence interval (CI) 0.88–0.97]. Complex procedures occurred in 37.4% and were associated with longest fibrosis length, lead calcification, dwelling time, and systolic heart failure. A fibrosis length of >30 mm predicted complex procedures (AUC 0.72; 95% CI 0.64–0.81).

**Conclusion:**

Pre-procedural CT allows accurate identification of high-risk anatomical features, particularly fibrosis length and calcifications, which independently predict both complicated and complex rotational mechanical TLE. These findings support the integration of CT imaging into procedural planning and individualized risk stratification.

## Graphical Abstract

**Graphical Abstract:**
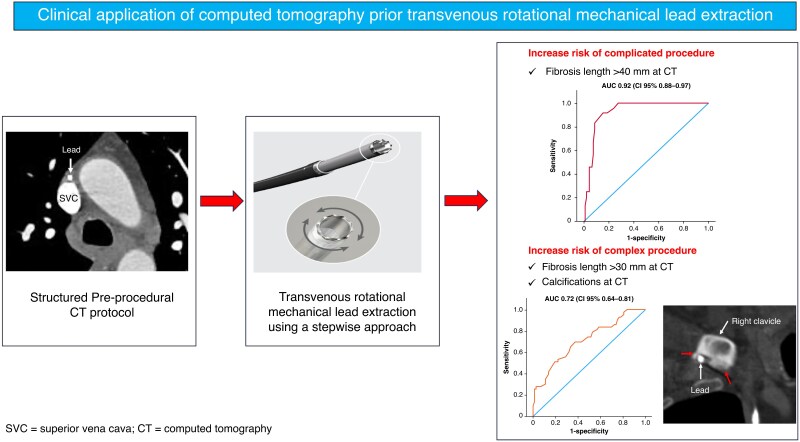

What’s new?

- In patients undergoing rotational mechanical sheath lead extraction, pre-procedural computed tomography allows for accurate identification of high-risk anatomical features.
- Fibrosis length and calcifications independently predict procedural complexity and complications.
- These results highlight the growing role of advanced imaging in tailoring transvenous lead extraction strategies to individual anatomical risk, supporting safer and more effective procedures.

## Introduction

Despite recent improvements, transvenous lead extraction (TLE), as a part of an overall lead management strategy, is still a challenging procedure, and it is associated with potential life-threatening complications.^1–3^ Several well-established patient- and lead-related factors—such as female sex, prolonged lead dwelling time, passive fixation mechanisms, and dual-coil defibrillator leads—are known to influence TLE outcomes.^3^ However, these may represent a surrogate for a more direct variable such as fibrosis, which directly leads to increased difficulty in TLE, failure, and feared complications such as superior vena cava (SVC) tears. Chronically implanted leads develop fibrous adhesions around surrounding structures and thus require different extraction sheaths, such as mechanical and laser sheaths.^1–3^ Computed tomography (CT) provides a way to visualize the leads as they course through the veins and into the cardiac chambers and may be able to identify fibrotic tissue and venous stenosis that may aid in the procedure planning.^4–11^ Previous studies have shown that cardiac CT can identify difficult lead extractions measured by fluoroscopy and procedural time, laser sheath size, a combination of laser and mechanical tools, and need of femoral snares. Major limitations of these studies include the absence of a standardized extraction protocol and variability in operator technique preferences, which may influence outcomes. Furthermore, the potential use of pre-procedural CT in patients undergoing rotational mechanical lead extractions as a first-line tool—rather than as an adjunct or alternative to the laser sheath to reduce the need for multiple devices—has not been previously evaluated. In recent years, the safety and effectiveness of the bidirectional rotational technique as a first-line tool for the extraction of chronically implanted leads have been established in large-scale, retrospective, and prospective multicentre experiences.^3,12–15^ Herein, the aim of this study was to assess if pre-procedural CT could identify high-risk operative features and predict increased procedural complexity in patients undergoing bidirectional rotational mechanical TLE as a first-line tool, using a stepwise approach with the available extraction tools.

## Material and methods

### Study population

In this retrospective single-centre study, patients undergoing transvenous rotational mechanical lead extraction by exclusively using the Evolution RL rotational sheaths with different mechanical ancillary tools (Cook Medical, Bloomington, IN, USA), supporting the procedure from September 2018 to May 2025, and who had a pre-procedural chest CT scan with lead extraction protocol were identified and included in the study. We did not employ any other lead extraction tool, including laser technique. Patient data including medical history, age, height, weight, body mass index (BMI), and procedural duration, as well as data including type of device, number of leads removed, CT findings, and complications, have been collected. Exclusion criteria included patients (i) aged <18 years, (ii) with recently implanted leads (implant duration < 12 months), and in whom leads had been explanted by simple traction without a locking stylet or Bulldog Lead Extender (Cook Medical). Furthermore, patients with incomplete CT examinations or CT without a lead extraction protocol including contrast injection (due to severe chronic kidney disease/not on dialysis) and those with severe contrast allergy or with atrial fibrillation with uncontrolled ventricular rates, which would not allow for proper ECG gating given very rapid heart rates, were excluded from the study. The study was conducted in compliance with the principles outlined in the Declaration of Helsinki and approved by the local medical ethics committee. The device manufacturer did not sponsor or influence the study in any way.

### Lead extraction protocol

All procedures were performed by two electrophysiologists experienced in TLE in the operating room, hybrid room, or electrophysiology laboratory under general anaesthesia or sedation with continuous electrocardiographic monitoring of arterial blood pressure. Standby cardiac surgery was always available. The cardiac surgeon was a designated operator familiar with the management of the lead extraction procedure and complications. In patients dependent on bradycardia support, a temporary pacemaker (PM) was inserted through the femoral vein. If present, the active fixation mechanism was retracted and manual traction was attempted. If this was unsuccessful, manual traction was attempted again using a locking stylet (Liberator, Cook Medical). When manual traction was ineffective, fibrous adhesions surrounding the lead were dissected using the Evolution RL rotational sheaths with available extraction tools (Evolution Shortie RL, One-Tie Compression Coil, and SteadySheath Evolution tissue stabilization sheath; Cook Medical) with a stepwise approach as previously reported in detail^16^ in all procedures. Free-floating leads and remnants after extraction were snared via a right jugular or femoral approach depending on how the leads were positioned using a Needle’s Eye Snare (Cook Medical). All patients were monitored for procedure-related complications at the time of extraction during their hospital stay. According to the protocol of our department, routine transthoracic echocardiography (TTE) is mandatory before TLE and after the procedure before discharge in all patients for the evaluation of underlying cardiac disease, including right and left ventricular systolic function and abnormalities of the valves, with particular attention to the tricuspid valve (TV) function.^17^ Additionally, intraprocedural transesophageal Echocardiogram (TEE) was also performed in all cases. Success and failure were defined according to the definitions of the 2017 Heart Rhythm Society and the 2018 European Heart Rhythm Association expert consensus.^1,2^ The complete procedural success rate, clinical success rate, and lead removal with clinical success rate were reported. For each removed lead, efficacy was determined as complete or incomplete lead removal.^1,2^ Complete lead removal was defined as lead explant or extraction with removal of all targeted lead material. Incomplete lead removal was defined as lead explant or extraction where part of the lead remains in the patient’s body (vascular or extravascular); failure was defined as removal when >4 cm length of the lead was abandoned after a removal attempt.^1,2^ Major complications were defined as outcomes that were life-threatening, resulted in significant or permanent disability or death, or required surgical intervention, and minor complications were defined as events related to the procedure that required medical intervention or minor procedural intervention.

### Computed tomography image acquisition and data collection

**Figure 1:**
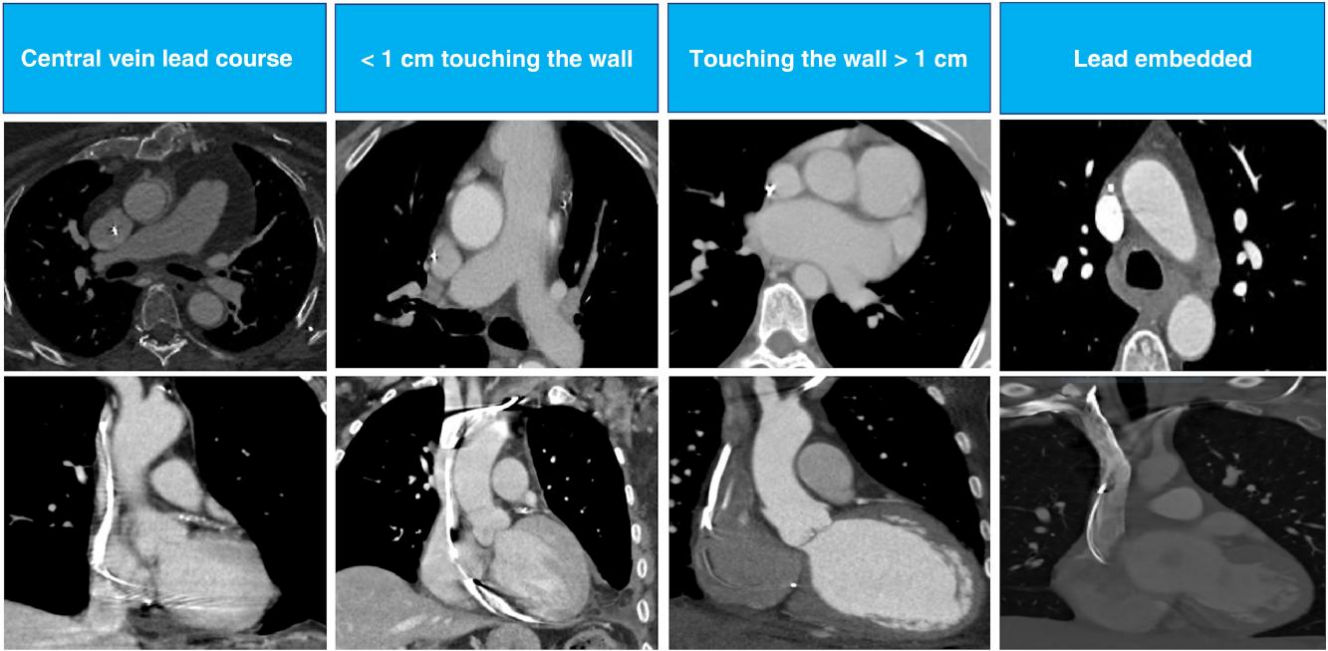
Cross-section CT scan showing the three groups in the study. Axial view and coronal reformatted images are represented for each group. Group 1: central vein lead course or < 1 cm touching the wall; Group 2: touching the wall > 1 cm; Group 3: lead embedded. CT, computed tomography.

All CT examinations were conducted using a 320-slice CT scanner with 0.5 mm detector rows (Aquilion ONE ViSION Edition; Toshiba Medical Systems, Otawara, Japan). The gantry rotation time was 350 ms, with automatic exposure control (SURE Exposure 3D, Toshiba/Canon Medical Systems) set to a standard deviation (SD) of 150 for non-contrast images and 110 for contrast-enhanced images. The scans were acquired using a 512 × 512 matrix, with slice thicknesses of 0.5 and 0.25 mm increments, utilizing kernel FC03 and iterative reconstruction (AIDR3D standard, Toshiba/Canon Medical Systems). As part of the structured TLE protocol, intravenous contrast (Iomeron® 400 mg/mL; Bracco Imaging Italy s.r.l., Milan, Italy) was administered at a dose of 60–80 mL with a flow rate of 3–4 mL/s adjusted for patient BMI, followed by 60 cc of saline solution. No scans without contrast were being acquired. The contrast-enhanced scan covered the entire extent of the chest and included a dose-modulated prospective ECG gating with image acquisition in end-diastole (centred at 75% of the R-R interval). The exam was completed with an untriggered venous phase scan, both for better visualization of the entire venous system and for the application of metal artefact reduction software (Single Energy Metal Artifact Reduction algorithm, SEMAR, Toshiba/Canon), which, in the version of our equipment, is not usable on ECG-gated scans. The post-processing included coronal, sagittal, and curved multiplanar reformats (MPR), in addition to the source axial images. The lead entrance site within the left or right subclavian vein or axillary vein and the lead course through the subclavian vein, brachiocephalic vein, and SVC, as well as the right atrium (RA), right ventricle (RV), or coronary sinus (CS), were all recorded, as was the integrity of each lead. According to previous studies,^8,9^ the fibrosis score was used to describe the adherence of the lead relative to the lumen and wall of the vessel and scored on a scale of 1–3 with 1 indicating lead within the contrast-enhanced lumen of the vessel without contact with the vessel wall (central course) or lead within the contrast-enhanced lumen of the vessel that abuts the wall of the vessel for a length of <1 cm, 2 indicating lead within the contrast-enhanced lumen of the vessel that abuts the wall of the vessel for a length of >1 cm, and 3 indicating lead that is outside the contrast-enhanced lumen of the vessel (led embedded); see *Figure 1*. These scores were evaluated in three zones of interest: Zone 1 from the lead entrance in the venous system to the brachiocephalic/SVC intersection, Zone 2 from the brachiocephalic/SVC interaction to where the shadow of the RA enlarges from the vertical lateral wall of the SVC, and Zone 3 from where the RA enlarges from the vertical lateral wall of the SVC to the end of the lead. Fibrosis length was measured in each zone, and the longest length was identified and correlated with outcomes.

Additional analyses on CT scans included stenosis of the vessel, calcifications, anomalous anatomy, thrombus adherent to leads, and perforations of the lead tip. Lead perforation was defined as lead tip extension into the epicardial fat seen with confidence in the cardiac phase with the least motion artefact. Venous occlusion was determined by the lack of the contrast lumen seen surrounding transvenous leads and the presence of collateral veins. Lead-associated calcifications were assessed by careful windowing of images to separate the density of calcifications along leads from lead metallic density. All cardiac CT scans were reviewed by two board-certified cardiac radiologists (R.M. and V.P.). These radiologists were blinded to the procedural outcomes of the patients undergoing TLE including success or failure of extraction, procedural time, and procedural complexity.

### Outcome analysis

Two distinct procedural outcomes were evaluated:

- The ‘complicated procedure’ outcome, defined as the occurrence of at least one of the following events: major complication, incomplete procedure, or use of a snare device. This composite endpoint was selected to capture clinically significant adverse events reflecting procedural safety and efficacy.
- The ‘complex procedure’ outcome, defined by the use of at least one of the following technical adjuncts: snare device or SteadySheath Evolution tissue stabilization sheath (Cook Medical). This outcome represents procedural difficulty and the requirement for additional extraction tools.

Both outcomes were assessed independently to provide complementary information regarding procedural risk and complexity. The use of a snare was included in both outcomes, reflecting its dual role as both a marker of procedural complexity and a component of procedural complications. The potential interdependence between the outcomes was taken into account during statistical analysis and interpretation.

### Statistical analysis

Continuous variables were reported as mean ± SD or as median [interquartile range (IQR), 25th–75th percentile] if normally or non-normally distributed, respectively. Categorical variables were presented as counts and percentages. The Shapiro–Wilk *W* test was used to assess the normality of continuous variables. Categorical differences between two groups were evaluated by using the *χ*^2^ test or Fisher’s exact test as appropriate. Comparisons of two groups of numerical variables were performed using Student’s *t*-test or the Wilcoxon rank-sum test as appropriate. The cohort was divided into three groups according to lead location within the SVC. Comparisons among the three groups were performed using the *χ*^2^ test for categorical variables and ANOVA or the Kruskal–Wallis test for continuous variables as appropriate according to their distribution. Univariable and multivariable analyses were used to identify factors associated with a worse outcome or procedural difficulty (associated with both aforementioned procedural outcomes). Receiver operating characteristic (ROC) curve analysis was performed to assess the discriminative ability of the longest fibrosis length in predicting both outcomes. The optimal cut-off value was determined using the Youden index, defined as the point maximizing the sum of sensitivity and specificity. The area under the curve (AUC) and the corresponding 95% confidence interval (CI) were reported. A two-tailed *P* < 0.05 was considered statistically significant. All analyses were performed using the SPSS statistical software package (version 29.0.2.0; SPSS Inc., Chicago, IL, USA).

## Results

### Study population

The study population comprised 115 patients [73% male; mean age 63 ± 15.5 years, median 64 (55–75)]. Patient and lead characteristics are reported in *Table 1*. Indications for TLE included infection in 59 cases (51.3%), lead malfunction in 41 (35.7%), lead-related tricuspid regurgitation in 4 (3.5%), system upgrade in 3 (2.6%), venous occlusion in 1 (0.9%), and others in 7 (6%). Patients with device infection were treated according to current guidelines and infectious disease consultation.

**Table 1:** Baseline clinical, device, and lead characteristics of the overall study population (*n* = 115)

Age (years)	63 ± 15.5
Male	84 (73%)
BMI (kg/m^2^)	25.4 ± 6.5
BMI < 25 kg/m^2^	51 (44.3%)
Hypertension	64 (55.6%)
Diabetes mellitus	32 (27.8%)
Chronic kidney disease (GFR < 30 mL/min)	15 (13%)
Prior cardiac surgery	28 (24.3%)
Ischaemic cardiomyopathy	18 (15.6%)
Congenital heart disease	8 (6.9%)
LVEF (%)	46.1 ± 15.3
LVEF < 35%	22 (19.1%)
Type of CIED prior to extraction	
PM	38 (33%)
ICD	43 (37.4%)
CRT-P	5 (4.3%)
CRT-D	26 (22.7%)
VDD	3 (2.6%)
Indication for extraction	
Infection	59 (51.3%)
Lead malfunction	41 (35.7%)
Lead-related TR	4 (3.5%)
System upgrade	3 (2.6%)
Venous occlusion	1 (0.9%)
Others	7 (6%)
Leads	
Dwelling time (months)	95 ± 73
Number of target leads	215
Passive fixation leads	103 (47.9%)
Median number of leads extracted	2 (1–2)

Values are median (IQR), *n* (%), or mean ± SD.

BMI, body mass index; CIED, cardiac implantable electronic device; CRT, cardiac resynchronization therapy; GFR, glomerular filtration rate; ICD, implantable cardioverter defibrillator; IQR, interquartile range; LVEF, left ventricular ejection fraction; PM, pacemaker; SD, standard deviation; TR, tricuspid regurgitation.

### Cardiac computed tomography findings

**Figure 2:**
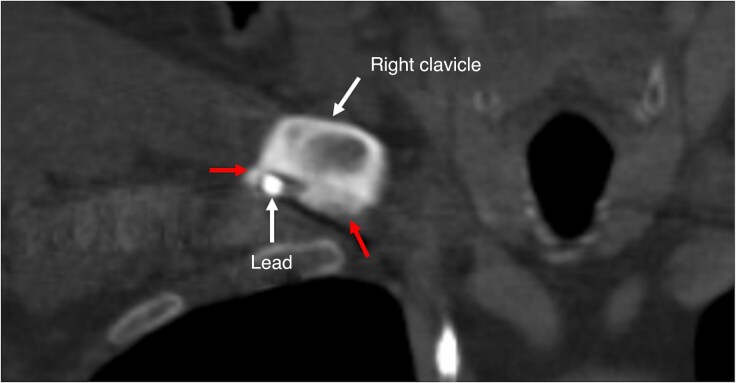
Computed tomography findings in the right costoclavicular space. The lead is in contact with the bone with calcifications (red arrows).

Baseline and procedural characteristics according to the fibrosis score are summarized in *Table 2*. No statistically significant differences were observed among the three fibrosis groups with respect to baseline patient characteristics, lead features, procedural parameters, use of powered sheaths, or procedural outcomes. The frequency and anatomical distribution of fibrosis, venous occlusion, lead-associated calcification, lead thrombosis, lead fracture, and perforation detected by CT are detailed in *Table 3* and *Figure 2*.

**Table 2:** Baseline and procedural characteristics according to the fibrosis score

	Central location or <1 cm touching the wall (*n* = 17)	Touching the wall >1 cm (*n* = 80)	Outside vein contour (lead embedded) (*n* = 18)	*P* value
Age (years)	67.1 ± 14	62 ± 16	60 ± 15.7	0.267
Male	12 (70.6%)	60 (75%)	12 (66.7%)	0.825
Diabetes mellitus	4 (23.5%)	24 (30%)	4 (22.2%)	0.377
GFR < 30 mL/min	2 (11.8%)	11 (13.7%)	2 (11.1%)	0.962
BMI < 25 kg/m^2^	8 (47%)	36 (45%)	7 (38.9%)	0.774
LVEF < 35%	4 (23.5%)	17 (21.2%)	1 (5.6%)	0.41
Previous cardiac surgery	5 (29.4%)	18 (22.5%)	5 (27.8%)	0.855
Ischaemic cardiomyopathy	2 (11.8%)	12 (15%)	4 (22.2%)	0.806
Infection	10 (58.8%)	41 (51.2%)	8 (44.4%)	0.640
Congenital heart disease	0 (0%)	5 (6.2%)	3 (16.7%)	0.26
Device types				
PM	6 (35.3%)	31 (38.7%)	6 (33.3%)	0.832
ICD	10 (58.8%)	47 (58.7%)	12 (66.7%)	0.774
Dual-coil ICD leads	4 (23.5%)	16 (20%)	3 (16.7%)	0.917
Passive fixation	12 (70.6%)	49 (61.2%)	11 (61.1%)	0.755
Abandoned leads	1 (5.9%)	11 (13.7%)	1 (5.6%)	0.624
Dwelling time (months)	89.3 ± 80	97.6 ± 76	88.9 ± 56	0.819
Major complications	1 (5.9%)	6 (7.5%)	0 (0%)	0.595
Minor complications	6 (35.3%)	11 (13.7%)	1 (5.6%)	0.081
Extraction tools >2	13 (76.5%)	67 (83.7%)	17 (94.4%)	0.491
Powered mechanical sheath use	13 (76.5%)	67 (83.7%)	17 (94.4%)	0.491
Use of a snare	2 (11.8%)	6 (7.5%)	0 (0%)	0.556
Procedural time (min)	132 ± 63	138 ± 81	104 ± 31	0.455
Complete procedural success	14 (82.3%)	69 (86.2%)	17 (94.4%)	0.713
Clinical procedural success	16 (94.1%)	75 (93.7%)	17 (94.4%)	0.976
Procedural failure	0 (0%)	6 (7.5%)	1 (5.5%)	0.685
Tricuspid valve regurgitation	4 (23.5%)	12 (15%)	2 (11.1%)	0.484

Values are median (IQR), *n* (%), or mean ± SD.

BMI, body mass index; GFR, glomerular filtration rate; ICD, implantable cardioverter defibrillator; IQR, interquartile range; LVEF, left ventricular ejection fraction; PM, pacemaker; SD, standard deviation.

**Table 3:** Cardiac CT findings

Adherence of the lead relative to the lumen and wall of the vessel at CT	Zone 1	Zone 2	Zone 3
Central location or <1 cm touching the wall	6 (7.3%)	10 (10.3%)	3 (16.7%)
Touching the wall >1 cm	60 (73.2%)	71 (73.2%)	11 (61.1%)
Outside vein contour (lead embedded)	16 (19.5%)	16 (16.5%)	4 (22.2%)
Stenosis	3 (2.6%)	1 (0.9%)	0 (0%)
Thrombus	2 (1.7%)	3 (2.6%)	0 (0%)
Calcification	6 (5.2%) Costoclavicular space	2 (1.7%)	0 (0%)
Cardiac perforation	0 (0%)	0 (0%)	3 (2.6%)
Lead fracture	0 (0%)	1 (0.9%)	0 (0%)

Values are *n* (%).

CT, computed tomography.

### Procedural data

A total of 215 leads were treated [mean number 2 ± 1 per patient, median 2 (1–2), range 1–5]. Of the target extracted leads, 70 (32%) were RA leads, 45 (21%) were RV pacing leads, 23 (12%) were CS leads for cardiac resynchronization therapy, 3 (1%) were VDD leads, 5 (2%) were conduction system pacing leads, and 69 (32%) were implantable cardioverter defibrillator (ICD) leads (23 dual-coil and 46 single-coil ICD leads). The leads with a passive fixation were 103 (47.9%). The mean implant duration was 95 ± 73 months [median 79 (39–138)]. At least one bidirectional rotational mechanical sheath was required in 97 patients (84%), while in the remaining 18 patients (16%), lead extraction was completed using manual traction and a locking stylet. One hundred and ninety-eight of 215 (92.1%) leads were completely extracted, whereas incomplete removal was observed in 17 leads (7.9%). The complete procedural success rate, clinical success rate, and lead removal with clinical success rate were 87% (100/115), 93.9% (108/115), and 94% (203/215), respectively. Major complications related to the TLE procedure occurred in seven patients (6%): haemopericardium requiring prompt surgical drainage (*n* = 3) and TV damage requiring intervention (*n* = 4). No injury to the SVC occurred in any cases, and no procedure-related deaths were reported.

### Outcome

**Figure 3:**
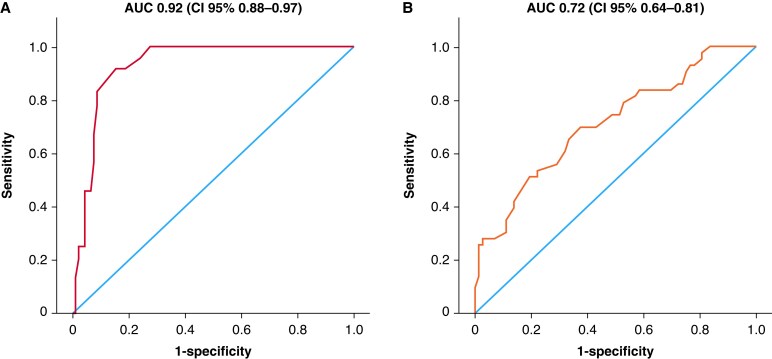
Receiver operating characteristic curve analysis on outcomes. (*A*) Complicated procedure according to the longest fibrosis length. (*B*) Complex procedure according to the longest fibrosis length. AUC, area under the curve; CI, confidence interval.

The procedure was classified as ‘complicated’ in 24 cases, defined by the occurrence of at least one of the following: major complication (*n* = 7), incomplete procedure (*n* = 15), or use of a snare device (*n* = 8). No significant differences were observed in baseline clinical or lead characteristics between patients with and without complicated procedures (*Table 4*). Patients experiencing complicated procedures more frequently underwent TLE for infection (*P* = 0.009) and had significantly longer procedural time (*P* = 0.007). Among CT findings, longest fibrosis length was significantly greater in patients with complicated procedures compared to those without (*P* < 0.001). No other CT parameters were significantly associated with procedural complications. Univariable logistic regression identified procedural time [odds ratio (OR) 1.00; 95% CI 1.001–1.010; *P* = 0.007], longest fibrosis length (OR 1.1; 95% CI 1.058–1.146; *P* < 0.001), and infection (OR 3.60; 95% CI 1.330–10.07; *P* = 0.012) as significant predictors of complicated procedures (*Table 6*). Multivariable analysis including these variables confirmed procedural time (OR 1.00; 95% CI 1.001–1.013; *P* = 0.028) and longest fibrosis length (OR 1.1; 95% CI 1.064–1.171; *P* < 0.001) as independent predictors. Longest fibrosis length was independently associated with each component of the composite endpoint: incomplete procedure (*P* < 0.001; OR 1.060; 95% CI 1.028–1.092), snare use (*P* = 0.001; OR 1.062; 95% CI 1.024–1.102), and major complications (*P* = 0.007; OR 1.047; 95% CI 1.012–1.083). Receiver operating characteristic curve analysis of fibrosis length yielded an AUC of 0.92 (95% CI 0.88–0.97) for predicting complicated procedure. A fibrosis length cut-off of 40 mm showed 95% sensitivity and 80% specificity (*Figure 3*).

**Table 4:** Baseline, lead, and clinical characteristics and CT findings of patients with and without a ‘complicated’ procedure

	‘Complicated’ procedure (*n* = 24)	No ‘complicated’ procedure (*n* = 91)	*P* value
Age (years)	65 ± 15	56 ± 14	0.247
Male	17 (70.8%)	67 (73.6%)	0.748
BMI < 25 kg/m^2^	13 (54.2%)	38 (41.8%)	0.276
Diabetes mellitus	6 (25%)	26 (28.6%)	0.728
GFR < 30 mL/min	3 (12.5%)	12 (13.2%)	0.929
Ischaemic cardiomyopathy	3 (12.5%)	15 (16.5%)	0.633
Previous cardiac surgery	5 (20.8%)	23 (25.3%)	0.652
LVEF ≤ 35%	7 (29.2%)	15 (16.5%)	0.160
Dwelling time (months)	115 ± 69	44 ± 58	0.057
Infection	18 (75%)	41 (45%)	0.009
Device types			
PM	7 (29.2%)	36 (39.6%)	0.349
ICD	16 (66.6%)	53 (58.2%)	0.527
Dual-coil ICD leads	6 (25%)	17 (18.7%)	0.491
Abandoned leads	5 (20.8%)	8 (8.8%)	0.097
Passive fixation	19 (79.2%)	53 (58.2%)	0.059
Procedural time (min)	189 ± 113	118 ± 54	0.007
CT findings			
Vein stenosis	0 (%)	4 (4.4%)	0.296
Vein occlusion	2 (8.4%)	10 (15.6%)	0.705
Calcification	3 (9.6%)	5 (5.5%)	0.230
Thrombus	2 (8.4%)	3 (3.3%)	0.282
Fibrosis score **≥** 2	18 (75%)	80 (87.9%)	0.113
Fibrosis length, Zone 1 (mm)	29 ± 24	23 ± 25	0.759
Fibrosis length, Zone 2 (mm)	48 ± 25	20 ± 19	0.303
Fibrosis length, Zone 3 (mm)	35 ± 20	18 ± 11	0.545
Longest fibrosis length (mm)	59 ± 21	24 ± 10	<0.001

Values are median (IQR), *n* (%), or mean ± SD.

BMI, body mass index; CT, computed tomography; GFR, glomerular filtration rate; ICD, implantable cardioverter defibrillator; IQR, interquartile range; LVEF, left ventricular ejection fraction; PM, pacemaker; SD, standard deviation.

The procedure was considered ‘complex’ in 43 cases, requiring at least one of the following: use of the SteadySheath Evolution tissue stabilization sheath (*n* = 41) or snare device (*n* = 8). Patients undergoing complex procedures had a higher prevalence of systolic heart failure (*P* = 0.019), longer lead dwelling times (*P* < 0.001), more frequent dual-coil ICD leads (*P* = 0.034), and longer procedural time (*P* = 0.004) (*Table 5*). Computed tomography findings revealed greater prevalence of calcifications (*P* = 0.002) and increased longest fibrosis length (*P* < 0.001) in this group compared to patients without complex procedures. No other CT parameters were significantly associated. Univariable logistic regression analysis demonstrated that procedural time (OR 1.0; 95% CI 1.001–1.008; *P* = 0.021), systolic heart failure (OR 3.00; 95% CI 1.168–7.880; *P* = 0.023), lead dwelling time (OR 1.0; 95% CI 1.005–1.018; *P* < 0.001), dual-coil ICD leads (OR 2.6; 95% CI 1.06–6.827; *P* = 0.038), calcifications (OR 13.00; 95% CI 1.635–116.558; *P* = 0.016), and longest fibrosis length (OR 1.1; 95% CI 1.020–1.066; *P* < 0.001) were significantly associated with complex procedures (*Table 6*). Multivariable analysis retained systolic heart failure (OR 5.3; 95% CI 1.549–18.327; *P* = 0.008), lead dwelling time (OR 1.0; 95% CI 1.002–1.017; *P* = 0.015), calcifications (OR 12; 95% CI 1.104–136.516; *P* = 0.041), and longest fibrosis length (OR 1.0; 95% CI 1.011–1.063; *P* = 0.05) as independent predictors. Receiver operating characteristic analysis of fibrosis length for complex procedure prediction resulted in an AUC of 0.72 (95% CI 0.64–0.81). A cut-off of 30 mm yielded 85% sensitivity and 54% specificity (*Figure 3*).

**Table 5:** Baseline, lead, and clinical characteristics and CT findings of patients with and without a ‘complex’ procedure

	‘Complex’ procedure (*n* = 43)	No ‘complex’ procedure (*n* = 72)	*P* value
Age (years)	62 ± 15	63 ± 16	0.423
Male	35 (81.4%)	49 (68.1%)	0.119
BMI < 25 kg/m^2^	19 (44.2%)	32 (44.4%)	0.978
Diabetes mellitus	11 (25.6%)	21 (29.2%)	0.678
GFR < 30 mL/min	5 (11.6%)	10 (13.8%)	0.728
Ischaemic cardiomyopathy	5 (11.6%)	13 (18.1%)	0.359
Previous cardiac surgery	11 (25.6%)	17 (23.6%)	0.812
LVEF ≤ 35%	13 (30.2%)	9 (12.5%)	0.019
Dwelling time (months)	128 ± 62	75 ± 72	<0.001
Infection	25 (58.1%)	34 (47.2%)	0.257
Device types			
PM	10 (23.2%)	33 (45.8%)	0.061
ICD	30 (69.8%)	39 (54.2%)	0.098
Dual-coil ICD leads	13 (30.2%)	10 (13.9%)	0.034
Abandoned leads	4 (9.3%)	9 (12.5%)	0.600
Passive fixation	31 (72.1%)	41 (56.9%)	0.104
Procedural time (min)	160 ± 82	115 ± 62	0.004
CT findings			
Vein stenosis	1 (2.3%)	3 (4.1%)	0.602
Vein occlusion	3 (7%)	9 (12.5%)	0.349
Calcification	7 (16.3%)	1 (1.4%)	0.002
Thrombus	2 (4.6%)	3 (4.2%)	0.902
Fibrosis score **≥** 2	42 (97.7%)	56 (77.8)	0.004
Fibrosis length, Zone 1 (mm)	29 ± 24	23 ± 25	0.759
Fibrosis length, Zone 2 (mm)	48 ± 25	20 ± 19	0.303
Fibrosis length, Zone 3 (mm)	35 ± 20	18 ± 11	0.545
Longest fibrosis length (mm)	55 ± 18	38 ± 20	<0.001

Values are median (IQR), *n* (%), or mean ± SD.

BMI, body mass index; CT, computed tomography; GFR, glomerular filtration rate; ICD, implantable cardioverter defibrillator; IQR, interquartile range; LVEF, left ventricular ejection fraction; PM, pacemaker; SD, standard deviation.

**Table 6:** Univariable and multivariable analysis

Predictors of complicated procedure				
	Univariable analysis		Multivariable analysis	
	OR (95% CI)	*P* value	OR (95% CI)	*P* value
Procedural time	1.0 (1.001–1.010)	0.007	1.0 (1.001–1.013)	0.028
Longest fibrosis length	1.1 (1.058–1.146)	<0.001	1.1 (1.064–1.171)	<0.001
Infection	3.6 (1.330–10.065)	0.012	3.8 (0.743–20.084)	0.108

Predictors of complex procedure				
	Univariable analysis		Multivariable analysis	
	OR (95% CI)	*P* value	OR (95% CI)	*P* value
Procedural time	1.0 (1.001–1.008)	0.021	—	
Longest fibrosis length	1.1 (1.020–1.066)	<0.001	1.0 (1.011–1.063)	0.005
Dual-coil ICD leads	2.6 (1.057–6.827)	0.038	—	
Calcification	13 (1.635–116.558)	0.016	12 (1.104–136.516)	0.041
LVEF < 35%	3.0 (1.168–7.880)	0.023	5.3 (1.549–18.327)	0.008
Dwelling time	1.0 (1.005–1.018)	<0.001	1.0 (1.002–1.017)	0.015

Odds ratios for fibrosis length are reported per 1 mm increase.

CI, confidence interval; ICD, implantable cardioverter defibrillator; LVEF, left ventricular ejection fraction; OR, odds ratio.

## Discussion

The increasing complexity and volume TLE procedures demand more refined tools for pre-procedural risk stratification to optimize outcomes and reduce the risk of complications. Our study evaluated the role of CT in patients undergoing TLE using rotational mechanical sheaths, focusing on the presence and extent of fibrosis as a predictor of procedural complexity and safety. Previous research studies have consistently identified lead dwelling time as a major risk factor for difficult and complicated TLE procedure. However, while dwelling time may serve as a surrogate for fibrotic encapsulation, it fails to capture individual anatomical variability and patient-specific fibrotic response. In contrast, cardiac CT offers a direct visualization and quantification of fibrous adhesions and calcifications, allowing a more personalized assessment of anatomical risk. In our cohort, longest fibrosis length measured on pre-procedural CT was found to be an independent predictor of both procedural complexity and complications, even after adjusting for traditional clinical risk factors such as dwelling time or infection. Notably, a fibrosis length of >40 mm demonstrated discriminative ability (AUC 0.92) for predicting complicated procedure, while >30 mm was associated with increased procedural complexity (AUC 0.72). These thresholds offer practical and actionable metrics for risk stratification and pre-procedural planning. Our findings are in agreement with and extend those of prior studies. The MILES study, a prospective multicentre trial, found that powered sheaths (laser or mechanical sheaths) were more frequently required in patients with higher fibrosis scores in the SVC zone.^9^ Similarly, Svennberg *et al.*^7^ and Patel *et al.*^8^ highlighted the relationship between CT-detected fibrosis, lead-associated calcifications, and increased procedural difficulty or the need to escalate extraction tools. However, those studies were limited by heterogeneous extraction approaches and variations in operator technique preferences, which may have influenced the outcomes.

The safety and effectiveness of rotating mechanical dilator sheaths have been described before.^3,11–16^ Laser sheaths are effective at handling fibrous lesions but can be less successful when confronted with heavily calcified lesions.^2^ Mechanical rotating dilator sheaths, on the contrary, can be more effective while cutting through and navigating densely calcified fibrotic lesions. In contrast to previous studies, which often included heterogeneous extraction techniques—such as combinations of laser, mechanical, and femoral approaches—our study focuses exclusively on extractions performed with rotational mechanical sheaths. This methodological consistency minimizes procedural variability and allows for a more precise assessment of the impact of fibrosis. Additionally, we introduce objective fibrosis length cut-offs that stratify the risk of procedural complications, providing actionable thresholds to support operator decision-making in clinical practice.

Importantly, calcifications, visualized on CT, also emerged as an independent predictor of procedural complexity. Unlike fibrosis, calcification is difficult or impossible to detect via fluoroscopy or chest radiography and may be a marker of particularly resistant adhesions. These results further support the notion that CT imaging offers unique and irreplaceable information in the pre-procedural planning phase, particularly in centres adopting laser sheaths where fibrotic and calcified lesions may present substantial challenges.^7–10^ Recently, Patel *et al.*^10^ emphasized the clinical relevance of CT-detected calcifications in patients undergoing TLE, demonstrating that the presence of lead-associated calcification or venous occlusion detected by CT increased the likelihood of crossover from laser to mechanical sheaths by five-fold and three-fold, respectively.

Imaging has become a cornerstone in the management of patients with complex arrhythmic substrates, providing valuable insights into cardiac anatomy and underlying structural abnormalities, as well as enabling the identification of catheter-related complications.^18^ Our findings further support the role of CT imaging not only in electrophysiological procedures but also in patients undergoing TLE. Moreover, our findings have potential clinical implications. Patients with a fibrosis length of >40 mm could be flagged pre-procedurally for extraction in a hybrid operating room, with immediate surgical backup available and greater allocation of resources. This strategy could minimize delays and improve outcomes in high-risk extractions.^19^ Future prospective studies should assess whether such CT-guided procedural planning results in measurable improvements in safety, efficacy, or resource utilization. Finally, our work may pave the way for further technological integration. Given the reproducibility of fibrosis length measurement, future efforts could explore automated quantification tools or artificial intelligence-driven risk scores, improving interobserver consistency and streamlining CT interpretation in busy clinical settings.

### Study limitations

Our study’s strengths include the exclusive use of rotational mechanical sheaths. However, limitations should be acknowledged. The retrospective single-centre design limits generalizability. Computed tomography scans were not performed on all patients undergoing TLE, which may introduce some selection bias of patients included in this study. While fibrosis length measurement was performed by experienced radiologists, interobserver variability was not formally assessed, and future studies should address this gap. Lastly, findings may not be directly extrapolable to centres using laser sheaths or other extraction techniques.

## Conclusions

In patients undergoing rotational mechanical sheath lead extraction, pre-procedural CT allows for accurate identification of high-risk anatomical features, including fibrosis length and calcifications, which independently predict procedural complexity and complications. This underscores the growing role of advanced imaging in tailoring TLE strategies to individual anatomical risk, supporting safer and more effective procedures. Integration of CT-derived parameters into routine pre-procedural planning—and prospective validation of fibrosis length cut-offs—is warranted to confirm and maximize their clinical utility.

## Data Availability

The experimental data used to support the findings of this study are available from the corresponding author upon request.
